# Effects of care pathways on the in-hospital treatment of heart failure: a systematic review

**DOI:** 10.1186/1471-2261-12-81

**Published:** 2012-09-25

**Authors:** Seval Kul, Antonella Barbieri, Erika Milan, Ilke Montag, Kris Vanhaecht, Massimiliano Panella

**Affiliations:** 1 Department of Biostatistics, Faculty of Medicine, University of Gaziantep, Gaziantep, Turkey; 2 Department of Clinical and Experimental Medicine, University of Eastern Piedmont 'A. Avogadro', Novara, Italy; 3 Santa Rita Hospital, Vercelli, Italy; 4 Center for Health Services and Nursing Research, School of Public Health, Catholic University, Leuven, Belgium; 5 European Pathway Association, Leuven, Belgium

**Keywords:** Care pathways, Heart failure, Hospitalisation costs, Length of hospital stay, Mortality rate, Readmission rate

## Abstract

**Background:**

Care pathways have become a popular tool to enhance the quality of care by improving patient outcomes, promoting patient safety, increasing patient satisfaction, and optimizing the use of resources. We performed a disease specific systematic review to determine how care pathways in the hospital treatment of heart failure affect in-hospital mortality, length of in-hospital stay, readmission rate and hospitalisation cost when compared with standard care.

**Methods:**

Medline, Cinahl, Embase and the Cochrane Central Register of Controlled Trials were searched from 1985 to 2010. Each study was assessed independently by two reviewers. Methodological quality of the included studies was assed using the Jadad methodological approach for randomised controlled trials, controlled clinical trials and the New Castle Ottawa Scale for case–control studies, cohort studies and time interrupted series.

**Results:**

Seven studies met the study inclusion criteria and were included in the systematic review with a total sample of 3,690 patients. The combined overall results showed that care pathways have a significant positive effect on mortality and readmission rate. A shorter length of hospital stay was also observed compared with the standard care group. No significant difference was found in the hospitalisation costs. More positive results were observed in controlled trials compared to randomized controlled trials.

**Conclusion:**

By combining all possible results, it can be concluded that care pathways for treatment of heart failure decrease mortality rates and length of hospital stay, but no statistically significant difference was observed in the readmission rates and hospitalisation costs. However, one should be cautious with overall conclusions: what works for one organization may not work for another because of the subtle differences in processes and bottlenecks.

## Background

Chronic heart failure (CHF) is a growing health problem. About 5.7 million people suffer from CHF in the United States [1] and approximately 5% of all acute medical admissions are HF-related in Europe [2]. HF is a leading cause of mortality. It has been stated that in 2006, 1 in 8.6 death certificates in the United States mentioned heart failure [3]. It is probably the most costly chronic disease. Its high prevalence and mortality rate places a huge economic burden on the health-care system. The estimated direct and indirect costs of HF in the United States in 2010 were $39.2 billion in total [3]. It was reported that the cost of HF care is two times higher than the cost of breast cancer, and three times higher than the costs of colorectal and lymphoma cancer care in the USA [4]. Thus, HF management is a big challenge for today’s health-care systems, and care services for HF need to be adjusted to reduce costs and mortality rates without compromising the quality of patient care. Care pathways (CPs) have become a popular tool to enhance the quality of care by improving patient outcomes, promoting patient safety, increasing patient satisfaction, and optimizing the use of resources [5,6]. The European Pathway Association (international not-for-profit association) defines CPs as “a complex intervention for the mutual decision making and organisation of care processes for a well-defined group of patients during a well-defined period" [7,8]. According to the European care pathway association, defining characteristics of CPs include: (i) An explicit statement of the goals and key elements of care based on evidence, best practice, and patients’ expectations and their characteristics; (ii) The facilitation of communication among team members and with patients and families; (iii) The coordination of the care process by coordinating the roles and sequencing the activities of the multidisciplinary care team, patients and their relatives; (iv) The documentation, monitoring, and evaluation of variances and outcomes; and (v) The identification of the appropriate resources. The aim of a care pathway is to enhance the quality of care, across the continuum, by improving risk-adjusted patient outcomes, promoting patient safety, increasing patient satisfaction, and optimizing the use of resources [7].

Although CPs have been used since the 1980s, and many high-quality articles have been published in international peer reviewed journals describing their benefits [9,10], the effectiveness of CPs is still controversial [11-13]. Systematic reviews and meta-analyses have received increasingly more attention from investigators for evaluating the effect of the implementation of CPs [14-16].

The results of some generic and disease-specific meta-analyses showed that pathways are effective in improving clinical and patient-related outcomes [14-19]. But none of them showed a significant effect of CPs on heart failure or in hospital treatment. Therefore, in this study we performed a disease-specific systematic review to show recent evidence of how CPs for hospital-based treatment of heart failure (HF) affect in-hospital mortality, length of in-hospital stay (LOS), rate of readmission, and hospitalisation costs.

## Methods

### Literature search

A dual approach was used to search the literature. Firstly, MeSH key words of "critical pathways" AND "heart failure" AND "congestive heart failure" were searched in Medline, Cinahl, Embase and the Cochrane Central Register of Controlled Trials databases. Secondly, a non-MeSH approach was used based on the following search string: ‘clinical pathway’ OR ‘critical pathway’ OR ‘care map’ OR ‘clinical path’ OR ‘multidisciplinary approach’ AND “heart failure OR congestive heart failure”. No limits were used except publication year. Because the first CPs in healthcare originated in the 1980s [20] the search was limited to years between 1985 and 2011. The authors of five relevant studies were contacted for further information. One author provided the original data [5]. The other authors did not reply [21-24].

### Study inclusion/exclusion criteria

We included randomised controlled trials (RCTs), controlled clinical trials (CCTs), controlled before-after studies (CBAs), and interrupted time series (ITS) in the systematic review. Studies were considered randomised when it was specifically stated in the text. All of the included studies compared the care provided through CPs with standard medical care. Studies were included when at least one of the following outcome indicators had been evaluated: hospital mortality rate, rate of readmission, length of hospital stay (the number of days of acute hospitalisation from admission to discharge) and hospitalisation costs (total cost of acute hospitalisation). Articles that were strictly descriptive (study protocols, review articles and theoretical articles), articles with no control group, articles that did not assess at least one of the four outcomes and articles for which the relevant information could not be retrieved were excluded. “Only studies which met the care pathways definition of EPA were included [8]. For this purpose a specific checklist has been developed to analyze the papers (Additional file 1: Annex 1). The papers that met at least 5 or 6 criteria have been included in the study (Additional file 1: Annex 2). All of the excluded articles and exclusion reasons were given in the Additional file 1.

### Outcome measures

In this study, results were combined according to four outcome indicators: hospital mortality rate, rate of readmission, length of hospital stay (the number of days of acute hospitalisation from admission to discharge) and hospitalisation costs (total cost of acute hospitalisation) to evaluate the overall effect of CPs on HF. These were the most commonly used and available outcomes in the included studies.

### Data extraction and quality assessment

The author, the publication year, the sample size, the characteristics of the population studied (age, sex, race, primary diagnosis, etc.), study design, type of control and outcome measures were recorded. Two reviewers independently screened the titles, abstracts and keywords to determine eligibility and assess methodological quality of the included studies and record the findings. The reviewers were blinded to the names of the authors, the institution where the study had been carried out, and the journal. Any disagreement was discussed with a third reviewer. Methodological quality of the included studies was evaluated using the Jadad methodological approach for RCTs and CCTs and the New Castle Ottawa Scale for interrupted time series [25,26]. All the studies that met the inclusion criteria (see above) but did not include any items of the checklists were excluded.

For dichotomous variables, counts were calculated by using percentages given in the articles. If an article only reported confidence intervals instead of standard deviations, standard deviations were calculated via the confidence interval formula (mean ± 1,96SE). Also, some results not described in text or tables but shown in graphs were extracted from the graphs by using data extraction software with the mean and confidence intervals given in the graph (XY data extraction software version 4.1).

### Data analysis

The primary meta-analyses were performed according to the guidelines set out by the Cochrane Collaboration regarding statistical methods [26]. For dichotomous variables, risk ratios (RR) and 95% confidence intervals (95% CI) were calculated using standard care group as the reference group. For the meta-analysis of continuous variables, the Weighted Mean Difference (WMD) with 95% CI was used. Since different kinds of study design were combined and the sample sizes of included studies were different, a ‘random effects model’ was used for all the outcomes [27]. In order to compare CPs with usual care, the costs were measured in United States dollars (US$) divided by 10,000 and expressed as the Weighted Mean Difference (WMD). One of the three included studies reported the costs in Euro (€) [10]. These costs were converted to US dollars ($) using the official exchange rate of the year of study data (year 2004). The costs were adjusted for the inflation rate in the United States and actualised to year 2004. Sensitivity analysis was performed to identify studies with a low quality [27]. For each outcome, meta analyses were repeated many times to check the robustness of findings using different assumptions. The largest, the smallest, the earliest studies and the studies with the most different results were excluded respectively to see the effect on the results. These sensitivity analysis results are given in the Additional file 1 [Supplementary-material S1]. In addition to sensitivity analyses, potential publication bias was assessed using a funnel plot which is a scatter plot of the estimate of the effect from each study in the meta-analysis against a measure of its precision, usually 1/SE [27]. The results for each outcome measure were presented separately for different study types.

The results for each outcome measure were presented separately for different study types with forest plots. Analyses were performed using the Review manager software package (RevMan), version 5.0 and a *P*-value smaller than 0.05 was considered to be statistically significant.

## Results

### Description of the studies

**Figure 1:**
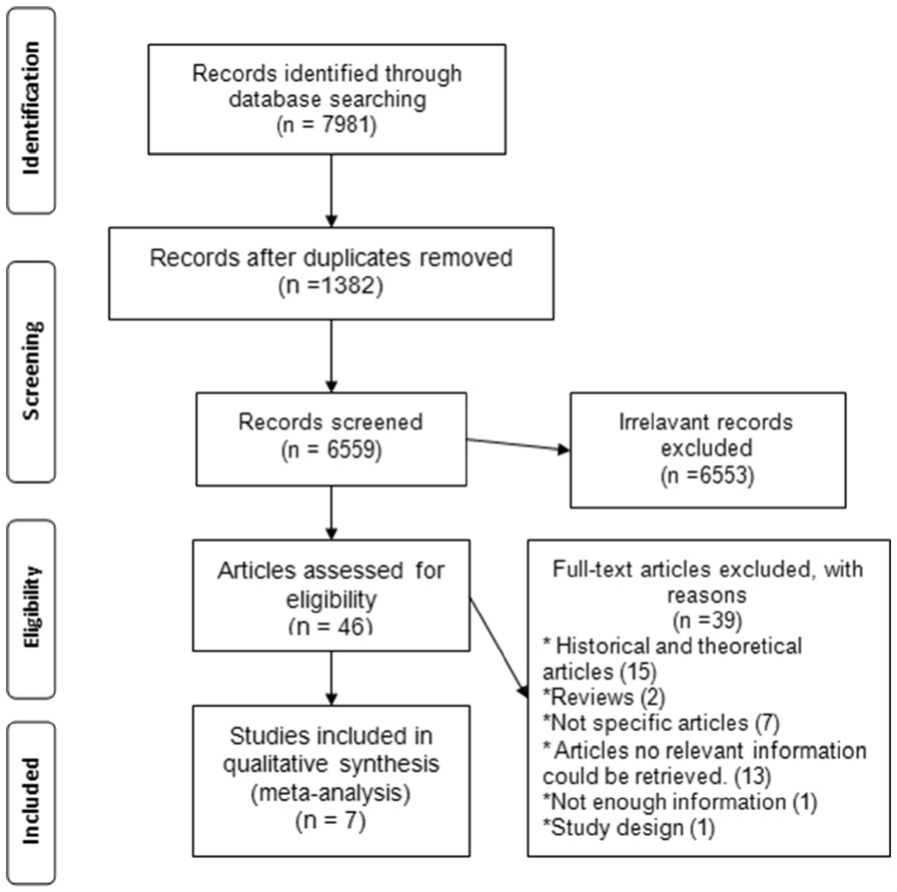
Flowchart of the selection of the studies.

The search strategy resulted in a total of 7981 records (6553 irrelevant, 1382 duplicated). Among 46 studies identified, only 7 publications met the study inclusion criteria [5,10,22,23,28-30] and they were included in the systematic review for a total sample of 3,690 patients, as shown in Figure 1. This systematic review consists of three randomised controlled trials [11,22,23] one interrupted time series [5] and three controlled studies [28-30].

Summary of included study characteristics were given in Table 1. Two publications reported results of studies with a multi-centre design, whereas five were single-centre studies. Two studies were based in Italy, four in the USA, one in Canada. The setting characteristics of the studies (hospital size, urban/rural typology, education, living conditions) were not fully reported.

**Table 1:** Summary of included study characteristics

**Author**	**Study design**	**Control type**	**Setting**	**Length of study (months)**	**Patients**	**Sex% Male**		**Mean age**		**Patients demographics**	**Main outcome**	**NYHA class**
						**Pathway**	**Control**	**Pathway**	**Control**			
Panella 2009	CRCT (multicentered)	Prospective	14 hospitals located in four Italian regions, Italy	19	429	47,7	51,2	81,7	79,7	Gender, age, severity at admission, co-morbidities	In-hospital mortality, length of stay, appropriateness of stay, unscheduled readmission rate	II,III,IV
Philbin 2000	CRCT (multicentered)	Prospective	10 Hospitals in Newyork, the USA	9	1504	49	48	77	74	Gender, age, race, insurance, medication use at admission	In-hospital mortality, length of stay, readmission rate, hospitalizations cost	-
Azad 2008	RCT (monocentric)	Prospective	Ottova Hospital, Canada	6	91	0	0	75,8	74,2	Gender, age, satisfaction scores	Mean hospitalization, In-hospital mortality	I,II,III
Panella 2003	Interrupted time series (monocentric)	Prospective	Italian hospital, Italy	12	246	43,62	-	78,9	-	Gender, age	In-patient mortality, length of stay, readmission rate within 31 days	II,III,IV
Discher 2003	Controlled trial (monocentric)	Historical	General hospital, the USA	12	593	-	-	-	-	-	Length of stay, hospitalization cost, Nursing satisfaction	-
Lanzieri 1999	Controlled trial (monocentric)	Historical	Central Maine Medical Central, Lewiston, the USA	18	73	53,8	55,9	68	65,7	Gender, age	In-hospital mortality, length of stay, readmission rate	-
Rauh 1999	Controlled trial (monocentric)	Historical	Elmhurst memorial hospital, Illnois the USA	12	754	42	50	75,8	74,39	Gender, age, co-morbidities	Length of stay, admission/readmission rate, hospitalizations cost	III,IV

### Effect of CPs: mortality rate

**Figure 2:**
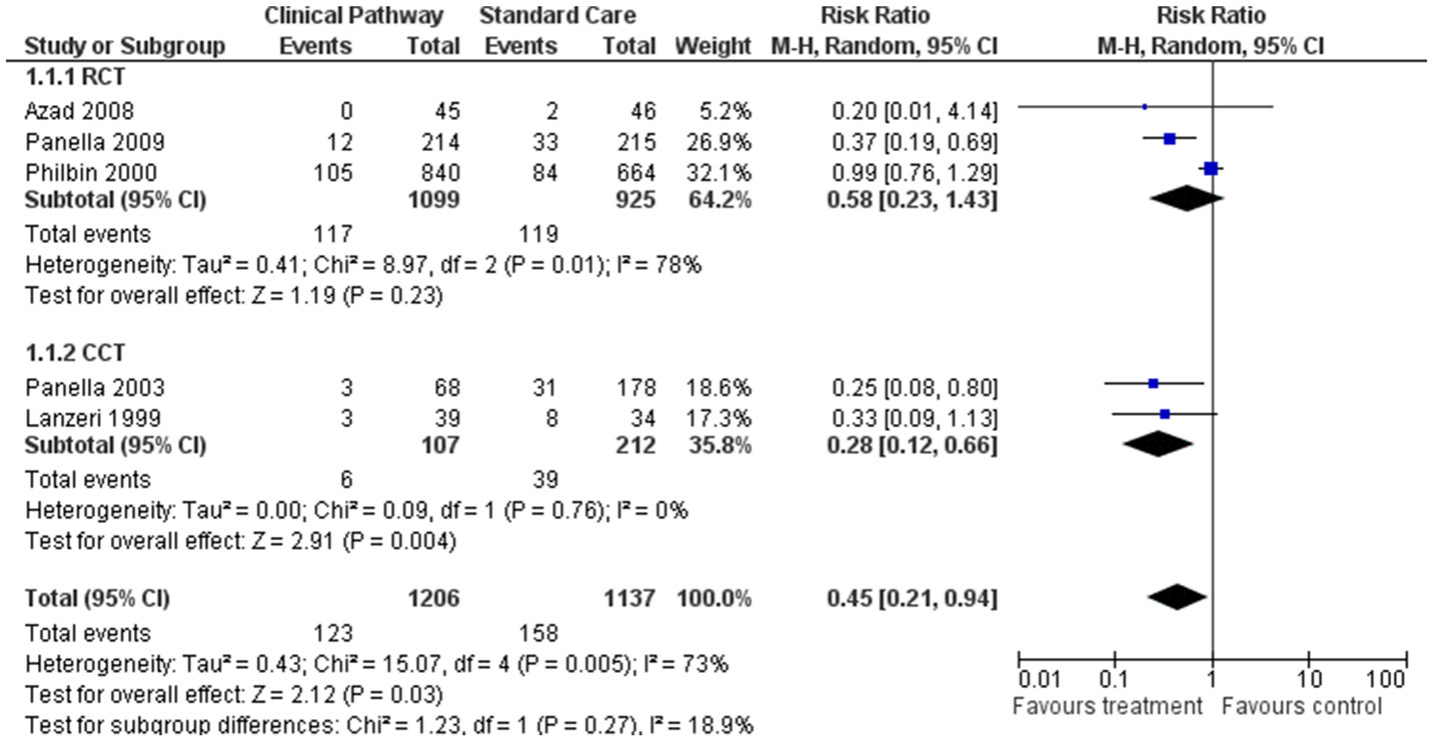
Forest plot of comparison for rate of hospital mortality.

Five studies were included in the systematic review (three randomised controlled, two controlled studies) to estimate the pooled risk ratio for mortality of the care pathway group compared to standard care. A total of 2,343 patients were analysed, including 1206 patients in the care pathway and 1137 patients in the standard care groups. The use of CPs had a significant positive effect in 2 of 5 studies, including the mortality rate [5,10]. Also, in the other three studies, the mortality rate decreased in the care pathway group, albeit not significantly [22,23,29]. Risk ratio (RR) estimates and their 95% confidence intervals are shown in Figure 2. The overall result of combining the RCTs was not statistically significant. The pooled risk ratio for mortality was 0.45, 95%CI *=* 0.21-0.94, *P =* 0.03.

### Effect of CPs: readmission rate

**Figure 3:**
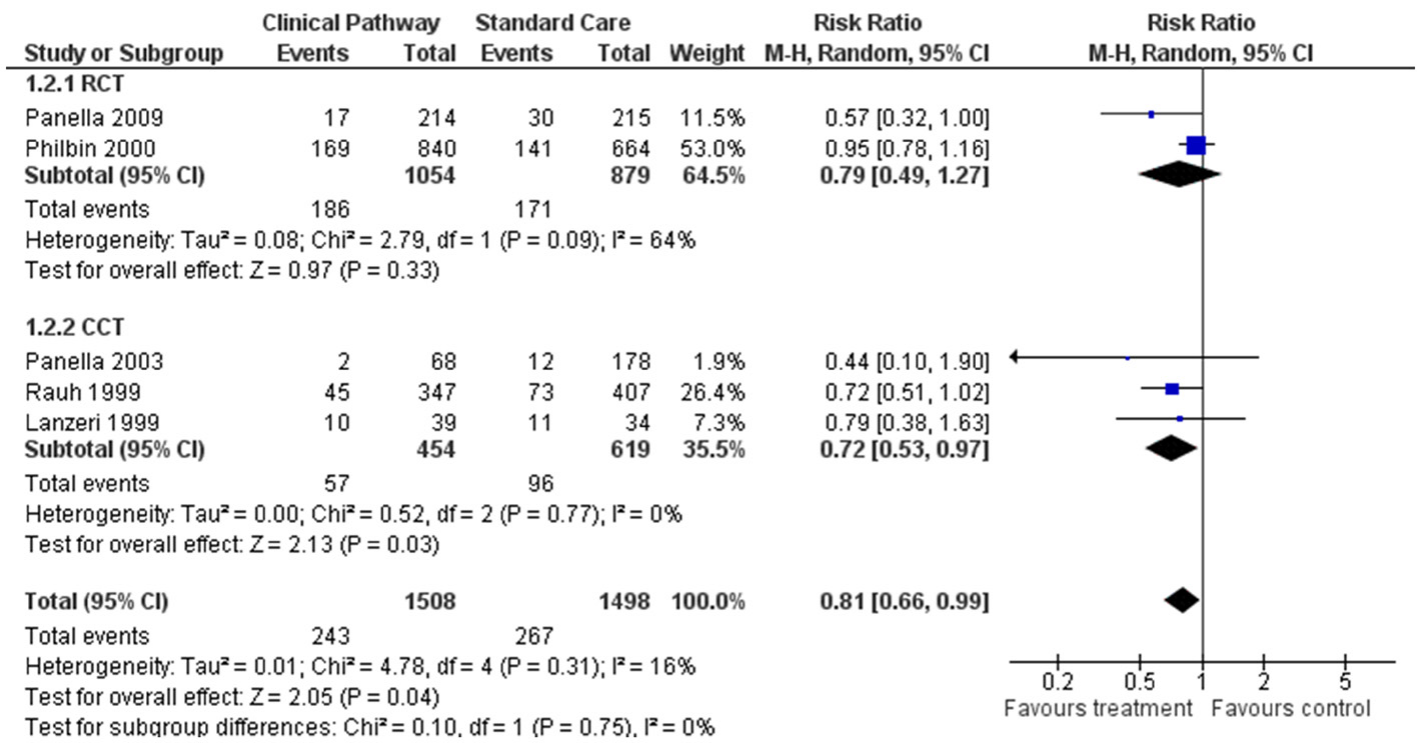
Forest plot of comparison for rate of readmission.

We included in the systematic review 5 studies reporting risk estimates for readmission rate [5,10,22,29,30]. A total of 3,006 patients were analysed, including 1508 patients in the care pathway and 1498 patients in the standard care groups. Figure 3 displays the result of the primary meta-analysis. One of the studies found that the risk of readmission significantly decreased in the care pathway group [10]. The overall RR for RCTs was 0.79, 95% CI = 0.49-1.27, P = 0.33. The overall result of meta-analysis was significant (RR = 0.81, 95%CI *=* 0.66-0.99, *P =* 0.04).

### Effect of CPs: LOS

**Figure 4:**
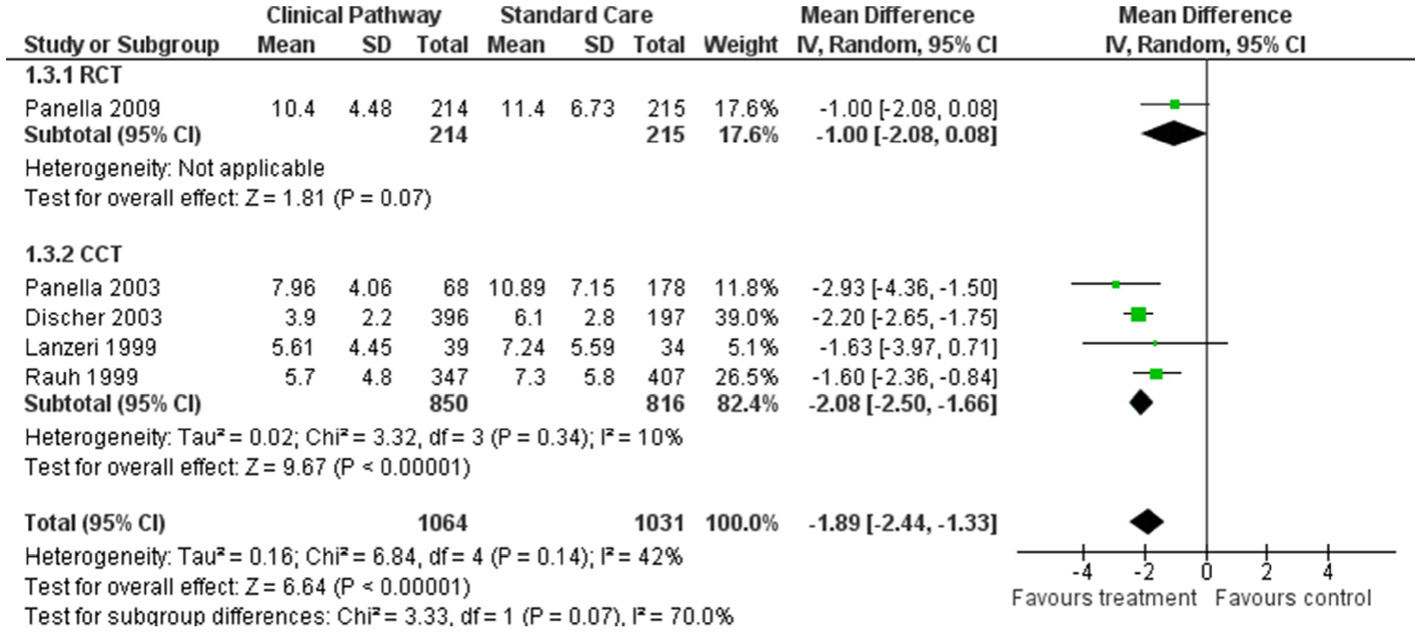
Forest plot of comparison for length of hospital stay.

Five studies were included in the systematic review (one randomised controlled, four controlled studies) representing a study population of 2,095 patients. Examining the effect of CPs on the length of stay, out of five studies [5,10,28-30] three studies showed a significantly shorter LOS in the care pathway group [5,28,30]. The overall results of the random-effects model showed that care pathway provided a positive reduction in LOS when compared with the standard care (WMD *= −*1.89, 95%CI *=* (−2.44-(−)1.33 days, *P* < 0.001) (Figure 4).

### Effect of CPs: hospitalisation costs

**Figure 5:**
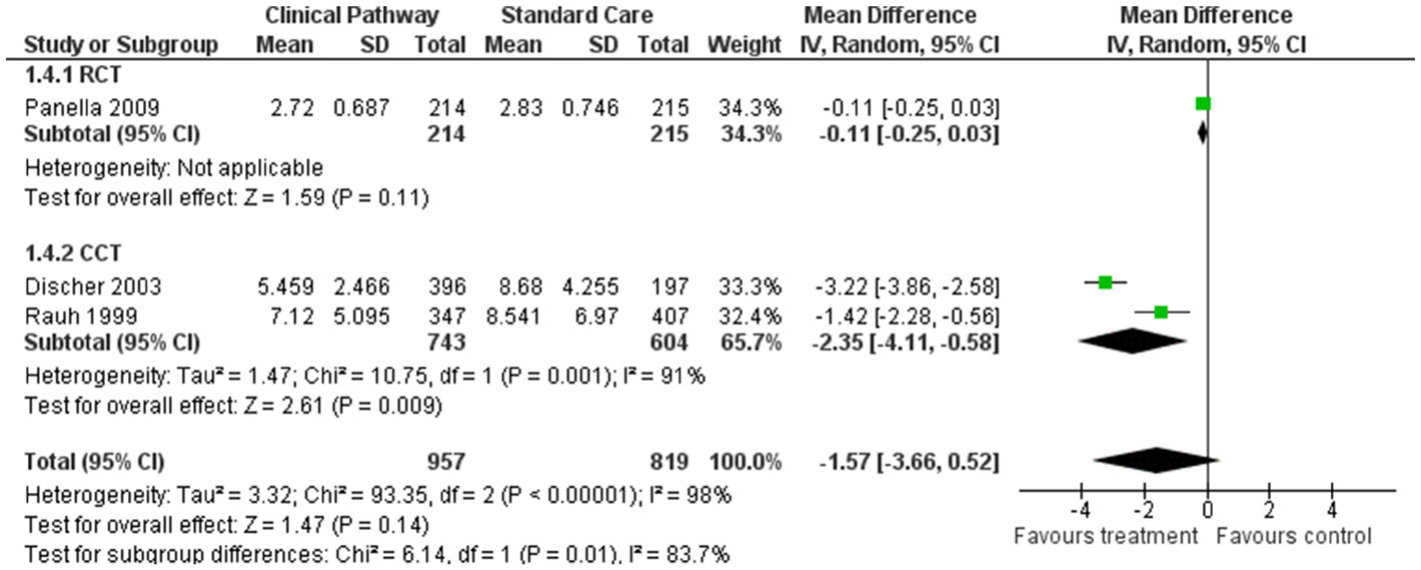
Forest plot of comparison for hospitalisation cost.

Three of the included studies (one randomised controlled, two controlled), representing a study population of 1776 patients, reported costs during hospitalisation [10,28,30]. Two of the CCTs [28-30] found significantly high hospitalisation costs for the care pathway groups. Also, the combined result of the controlled studies was significant (WMD *=* (−)2.35, 95%CI *=* (−)4.11-(−)0.58, *P* < 0.009) but the RCT was not (WMD *=* (−)0.11, 95%CI *=* (−)0.25-0.03, *P* = 0.11). Overall, the meta analysis results of the random effects model did not show any significant differences in hospitalisation costs when the CPs were compared with the non-pathway based care, as shown in Figure 5 (WMD *=* (−)1.57, 95%CI *=* (−)3.66-0.52, *P* = 0.14).

## Discussion

In this systematic review, the results of 7 publications which met the study inclusion criteria were combined to determine the effect of CPs on the outcomes of in-hospital treatment for heart failure: in-hospital mortality, rate of readmission, LOS and hospitalisation costs.

This study is the first systematic review to show an impact of CPs on the treatment of heart failure.

The primary finding of this systematic review was that the implementation of CPs for heart failure reduced hospital mortality and length of stay without increasing hospitalisation costs. Also readmission rate decreased in the care pathway groups (Figure 3). By looking at this data, it is evident that CPs can effectively improve the quality of care provided to patients suffering from heart failure. Lower mortality rates and shorter hospital stays among CP patients are associated with standardisation of the care process. However, we must be careful with the overall conclusions: pathways are not just documents recorded in the patient charts but a way to organize and standardise a multi-disciplinary care for patient groups using well-known quality improvement methodologies [31].

In the included studies, all patients were admitted to hospital with a primary diagnosis of chronic heart failure. The definition of readmission rate was different in the included studies. In two studies, readmission was recorded within 31 days after discharge [5,10]; in one study it was recorded within 90 days of discharge [30] and in two studies 6 months after hospital discharge [27,29]. For all included studies cost was calculated for hospital stay per-patient but no additional information was given about the cost calculation.

There are two previous generic meta-analysis reports suggesting that implementation of CPs can achieve a reduction in some of the patient outcomes [18,19]. Although some of the findings were confirmed by our study, there are also several reasons that preclude comparison of our findings with generic meta-analyses. Firstly, pathways are a learning tool both at professional (individual) and organizational (team) level [32], and pathways for different patient groups define different tasks to be learned, which affects performance of pathways and also patient outcomes. Also most of the outcomes such as mortality rate, readmission rate, LOS and costs of hospitalisation are related to types of treatments given to patients and show differences among specific patient groups. Different interventions are sometimes classified under the same term. When different clinical topics are included in the meta-analysis, the effect size of the intervention mostly shows huge variations between pathways for different patient groups. Exploring the reasons behind the heterogeneity rather than derivation of a single summary estimate of the effect size has emerged as the main goal of the meta-analysis [33]. Some statistics which were estimated for general populations from the generic meta-analysis such as weighted mean difference, OR or RR cannot be extrapolated to any patient group.

This systematic review has some limitations. We initially identified 46 relevant studies among 7981 total search results. However, only 7 studies could be considered as CPs and met our inclusion criteria. The scarcity of publications on CP probably results from the fact that CPs have only recently become a popular tool and the associated benefits for heart failure interventions are still not clear. Therefore, not many studies could be included to evaluate the effect of CPs on in-hospital treatment of heart failure.

In this systematic review, we observed that the findings of controlled trials were always more positive than the RCTs. In the included papers all of the study groups were experimental and the data was collected prospectively. Among included studies, three were randomized controlled clinical trials and one of them was an interrupted time series. In this study, measurements were performed on the same patients before and after the experiment, so patient characteristics are unlikely to have affected the outcomes. But 3 of the included studies were non-randomised; and historical controls were used. In the non-randomised studies, differences in patient characteristics may have affected the outcomes. Although patient characteristics were similar in the study and control groups (Table 1), differences in patient characteristics may explain more positive results in CCTs. Moreover, none of the studies mentioned in-hospital complications; thus, the association between hospital complications, costs and LOS could not be addressed. In addition, we assume that combining clinical indicators with a satisfaction survey could have given a more accurate measure of the true level of quality achieved through CPs [34]. Concerning included studies, only two studies measured patient satisfaction score [10,23] and reported no significant effect. As studies used different quality assessment tools, combining of results was not possible; so, patient satisfaction scores were not included in the systematic review as an outcome.

Since heart failure is defined in different ways and inclusion criteria can vary from study to study, individual patient data meta-analysis would have been a good option to overcome some of the shortcomings of our analysis. The New York Heart Association classification would have been used to perform subgroup analysis. Unfortunately only four of the seven studies reported NHYA classification and they did not provide separate results for each classification. Another limitation of our study was that despite performing a search from 1985 to 2011, we could not identify any eligible study until 1999. Thus, we cannot generalise our results for the last 26 years but our study results are valid for the last 13 years.

In 6 studies [5,10,23,28-30], pathway and control groups were well-balanced with regard to the number of the patients, and in 5 studies [5,10,22,29,30] pathway and control groups were age- and sex-matched. Moreover, these five studies were similar with respect to the age and sex ratio (female/male) of the patients. One study did not provide patient characteristics [28] and one study enrolled only female patients [23]. The title of this study suggests that the study sample consists of older women, but the mean age of the patients was similar to the other studies included.

Although the above mentioned patient characteristics of the studies were similar, we observed a high heterogeneity in the statistical analysis by use of the I^2^ statistic, which was introduced by Higgins et al. [27]. Although *I*^2^ values of 75% were described as high [27], no desired threshold for *I*^2^ was determined. Recently it was mentioned that heterogeneity is to be expected in a meta-analysis because studies are performed by different teams in different settings [34]. Using a random-effects model to reduce the effect of heterogeneity on a statistical analysis is a widely used approach [27]. In addition, it is known that sensitivity analysis should be accompanied by an analysis to show the effect of heterogeneity on statistical analysis [35]. A risk of bias check list has been developed according to pre-defined inclusion criteria and papers were ranged according to the risk of bias for each outcome. Results of the sensitivity analysis were given in the Additional file 1. In sensitivity analysis, relative risks were similar for in-hospital mortality, re-admission rate and length of stay. A significantly greater mean difference was observed for hospitalization cost which is probably a consequence of the small number of studies included for this outcome.

There is an international controversy on the definition of pathways [36-38]. Vanhaecht et al. defined 17 criteria [39] and the paper by De Bleser et al. gave a detailed overview on how a definition of pathways could be built [36]. Recently Kinsman et al. introduced a definition in their paper, consisting of five criteria for care pathways based on the aforementioned papers previously published [37]. Both criteria are valid and usable. In this study only studies meeting the CPs definition of EPA, which covers Kinsman et al. criteria’s, were included in this systematic review [6].

According to the definition, pathways describe patient processes and in the planning of a pathway, identification, and resolution of process ‘bottlenecks’ frequently occur. However, what works for one organization may not work for another, because of subtle differences in these processes and bottlenecks. Organizations are also different in their readiness for and capacity to change. These are often referred to as context issues, which influence implementation and effectiveness [40]. Hawe, Shiell, and Riley (2004) suggested as a possible solution to standardize complex interventions, the function and process of the intervention and not only the components [41]. This information on the context and the change process is critical to the ability of others to adapt the findings of a study to their own setting [40,42]. A pathway which works in one place may be ineffective in another without this key knowledge. In our opinion, this important issue did not affect the validity of our findings. In fact, all the papers that have been selected in our review included process and outcome indicators that provided data to understand if pathways worked (Additional file 1: Annex 2). Therefore we think that our paper addresses the research question adequately and we also think that our findings are based on consistent data.

A final remark is that the literature suggests that when researchers implement a pathway in a team that is already performing well, one may not identify significant improvements. A poorly performing team may, on the other hand, be greatly improved by the implementation of a new pathway, but these teams may not always be interested in improving the organization of their care process [31]. Pathways are one of the tools interdisciplinary teams can use to audit, standardise and improve the organisation of care.

## Conclusion

Our goal was to demonstrate the impact of care pathways on patient outcomes. By combining all possible results of care pathways, it can be concluded that care pathway for heart failure treatment showed a decreased mortality rate and length of hospital stay, but no statistically significant difference was observed in the readmission rates and hospitalisation costs. Although some findings of this systematic review showed that care pathways have a positive effect on some outcomes, one should consider the limitations of this study while interpreting the results. In the future, a meta-analysis should be performed with an increased number of included studies to decrease heterogeneity of study designs. We suggest that the findings and possible limitations of this study be considered in the planning of future meta-analyses to shed light on the effect of pathways in specific patient groups.

## Abbreviations

CCT: Controlled clinical trial; HF: Heart failure; LOS: Length of in hospital stay; MeSH: Medical subject headings; RCT: Randomised controlled trial; RR: Risk ratio; WMD: Weighted mean difference.

## Competing interests

The authors declare that they have no competing interests.

## Authors’ contributions

AB and EM searched for and selected the publications. SK and MP extracted and analysed the data. SK conceived of the study and wrote the first draft of the paper. KV and IM participated in the study design and its coordination and helped to write the final manuscript and discuss the results. All authors read and approved the final manuscript.

## Authors’ information

MP is the President of the European Pathway Association, E-P-A ( http://www.E-P-A.org) , KV is the Secretary General of E-P-A.

## Pre-publication history

The pre-publication history for this paper can be accessed here:

http://www.biomedcentral.com/1471-2261/12/81/prepub

## Supplementary Material

Additional file 1 **Supplemental material.** Supplement 1: Annex 1. Check list for CP definition of European Pathway association. Supplement 2: Annex 2. Check list for included studies. Supplement 3: List of excluded studies. Supplement 4: Annex 3. Check list for risk of bias for each included papers. Supplement 5: Result of sensitivity analysis. Click here for file
