# Supplementary material for: Effects of care pathways on the in-hospital treatment of heart failure: a systematic review
Source: BMC Cardiovasc Disord. 2012 Sep 25;12:81. doi: 10.1186/1471-2261-12-81 (PMC3507726; doi:10.1186/1471-2261-12-81)
Supplement: Additional file 1 — Supplemental material. Supplement 1: Annex 1. Check list for CP definition of European Pathway association. Supplement 2: Annex 2. Check list for included studies. Supplement 3: List of excluded studies. Supplement 4: Annex 3. Check list for risk of bias for each included papers. Supplement 5: Result of sensitivity analysis. [file 1471-2261-12-81-S1.docx]

**Supplemental material**

Supplement 1: Annex 1. Check list for CP definition of European Pathway association.

Supplement 2: Annex 2. Check list for included studies

Supplement 3: List of excluded studies

Supplement 4: Annex 3. **Check list for risk of bias for each included papers**

Supplement 5: Result of sensitivity analysis

Annex 1. Check list for CP definition of European Pathway association.

| **Question** | **Yes** | **No** |
| --- | --- | --- |
| Is the CP a complex intervention for the care processes for a well defined group of patients during a well defined period? |  |  |
| Does the CP describe the key elements of care based on evidence and best practice? |  |  |
| Does the CP organise the care processes by coordinating the roles and sequencing the activities of the multidisciplinary team? |  |  |
| Does the CP facilitate the communication among the team members and with patients and families? |  |  |
| Does the CP contemplate the documentation of outcome's monitoring and evaluation? |  |  |
| Dose the CP describe the appropriate resources? |  |  |

Annex 2. Check list for included studies

| **Question** | **Azad 2008** | **Discher 2003** | **Lanzieri 1999** | **Panella 2003** | **Panella 2009** | **Philbin 2000** | **Rauh 1999** |
| --- | --- | --- | --- | --- | --- | --- | --- |
| Is the CP a complex intervention for the care processes for a well defined group of patients during a well defined period? | 1 | 1 | 1 | 1 | 1 | 1 | 1 |
| Does the CP describe the key elements of care based on evidence and best practice? | 1 | 1 | 1 | 1 | 1 | 1 | 1 |
| Does the CP organise the care processes by coordinating the roles and sequencing the activities of the multidisciplinary team? | 1 | 1 | 1 | 1 | 1 | 1 | 1 |
| Does the CP facilitate the communication among the team members and with patients and families? | 0 | 1 | 1 | 1 | 1 | 0 | 0 |
| Does the CP contemplate the registration of outcome's monitoring and evaluation? | 1 | 1 | 1 | 1 | 1 | 1 | 1 |
| Dose the CP describe the appropriate resources? | 1 | 1 | 1 | 1 | 1 | 1 | 1 |

List of excluded studies

| **Study No** | **Author** | **Year** | **Title** | **Journal** | **Exclusion reason** |
| --- | --- | --- | --- | --- | --- |
| 1 | Andrews A | 2009 | Heart failure tools | Crit Pathw Cardiol. | Theoretical |
| 2 | Clancy TR | 2009 | Putting it altogether: improving performance in heart failure outcomes, part 2 | J Nurs Adm | Theoretical |
| 3 | Iannone P | 2009 | Effectiveness of a multipurpose observation unit: before and after study. | Emergency Medicine Journal | Not specific |
| 4 | McCue JD | 2009 | Quality toolbox: clinical pathways can improve core measure scores. | J Healthc Qual. | Not specific |
| 5 | Herzog E | 2009 | Novel pathway for sudden cardiac death prevention. | Crit Pathw Cardiol | Theoretical |
| 6 | Hill CA | 2009 | Acute heart failure: too sick for discharge teaching? | Crit Care Nurs | Theoretical |
| 7 | Yera-Casas AM | 2009 | Evaluation of an educational intervention in elderly patients with heart failure performed by nurses using a standardized care plan | Enferm Clin. 2009 | Not specific |
| 8 | Hadjistavropoulos HD | 2008 | Provider perceptions of implementation of integrated care pathways for patients with chronic heart conditions | J Cardiovas Nurs | Not specific |
| 9 | Peterson ED | 2008 | Implementing critical pathways and a multidisciplinary team approach to cardiovascular disease management | Am J Cardiol. | Theoretical |
| 10 | Gardetto NJ | 2008 | Critical pathway for the management of acute heart failure at the Veterans Affairs San Diego Healthcare System: transforming performance measures into cardiac care. | Crit Pathw Cardiol. | Theoretical |
| 11 | Panella M | 2007 | A cluster randomized controlled trial of a clinical pathway for hospital treatment of heart failure: study design and population | BMC Health | Theoretical |
| 12 | Panella M | 2005 | The effectiveness of an integrated care pathway for inpatient heart failure treatment: results of a trial in a community hospital. | Journal of Integrated Care Pathways | Theoretical |
| 13 | Herzog E | 2005 | Translation of critical pathways for acute coronary syndrome and for acute heart failure into admission forms and discharge planning | Crit Pathw Cardiol. | Theoretical |
| 14 | Dykes PC | 2005 | Clinical practice guideline adherence before and after implementation of the HEARTFELT (HEART Failure Effectiveness & Leadership Team) intervention. | J Cardiovas Nurs | Not specific |
| 15 | Ranjan A | 2003 | Effectiveness of the clinical pathway in the management of congestive heart failure | South Med J. | Review Article |
| 16 | Anonimo | 2003 | SSM's CARE PATHWAYS boost patient compliance, lower readmission rates | Hosp Case Manag | No relevant information |

| **Study No** | **Author** | **Year** | **Title** | **Journal** | **Exclusion reason** |
| --- | --- | --- | --- | --- | --- |
| 17 | Hahn J | 2002 | Integrating service excellence in a CHF clinical pathway pilot project. | J Healthc Qual. | Theoretical |
| 18 | Anonimo | 2001 | Voluntary CHF pathways cut costs, boost outcomes | Clin Reour Manag | No relevant information |
| 19 | Hoskins LM | 2001 | A clinical pathway for congestive heart failure (part 1) | Home Healthc Nurse | Not specific |
| 20 | Hoskins LM | 2001 | A clinical pathway for congestive heart failure (part 2) | Home Healthc Nurse | Theoretical |
| 21 | Graybeal K | 2001 | Heart failure management in a community hospital system | Lippincotts case Manag | Theoretical |
| 22 | Dahl J | 2000 | The effects of an advanced practice nurse-directed heart failure program | Nurse Practitioner | No relevant information |
| 23 | Kosnik L | 1999 | Treatment protocols and pathways: improving the process of care | Crit care nurse | No abstract |
| 24 | Cardozo L, Atherns S. | 1999 | Assessing the efficacy of a clinical pathway in the management of older patients hospitalized with congestive heart failure | J Healthc Qual. | Not enough information |
| 25 | Knox D,mischke L | 1999 | Implementing a congestive heart failure disease management program to decrease length of stay and cost | Cardiovasc nurse | Review Article |
| 26 | Warner PM | 1999 | Heart failure management | Journal of Nursing Administration | Theoretical |
| 27 | Anonimo | 1998 | CHF pathway moves from theoretical to practical: process approach finds hidden costs, lowers LOS | Disease State Management | No relevant information |
| 28 | Cardozo L, Atherns S. | 1998 | Implementing a clinical pathway for congestive heart failure: experience at a teaching hospital | Qual Manag Health Care. | No relevant information |
| 29 | Anonimo | 1998 | Outcomes analysis, clinical pathways improve care, cut cost | Exec solut healthc manag | No abstract |
| 30 | Anonimo | 1998 | Jefferson CHF pathways includes severity adjustment | Hosp Case Manag | No relevant information |
| 31 | Stegall GC | 1998 | Blueprints: a critical pathway alternative | Hosp Case Manag | No relevant information |
| 32 | Anonimo | 1998 | Continuum-focused CHF path cuts LOS to four days | Hosp Case Manag | No relevant information |
| 33 | Anonimo | 1997 | CHF program drastically cuts readmission rates... transitional care is key to results | Disease State Management | Theoretical |
| 34 | Anonimo | 1997 | Consortium benchmarks CHF, develops care path | Healthc Benchmarks | No relevant information |
| 35 | Jungkind K, shaffer R. | 1996 | CHF (congestive heart failure) path cuts length of stay, saves $2300 per case | Hosp Case Manag | No relevant information |
| 36 | Roy P, Janson E. | 1996 | CHF (congestive heart failure) path begins in ED, saves hospital $1000 per patient | Hosp Case Manag | No relevant information |
| 37 | Welsh C | 1996 | Congestive heart failure: a continuum of care | J Nurs care Qual | Theoretical |
| 38 | Balesky JR, Provenzano LM | 1995 | Collaborative development of a clinical pathways for congestive heart failure | [J Healthc Qual.](http://www.ncbi.nlm.nih.gov/pubmed/10153408) | Theoretical |
| 39 | Anonimo | 1994 | CHF pathways cuts annual pharmacy costs and per-case LOS | Disease State Management | No relevant information |

**Annex 3. Check list for risk of bias for each included papers**

| **Criteria** | **Azad**  **2008** | **Discher**  **2003** | **Lanzieri**  **1999** | **Rauh**  **1999** | **Panella**  **2003** | **Philbin**  **2000** | **Panella**  **2009** |
| --- | --- | --- | --- | --- | --- | --- | --- |
| **Meeting all of EPA criteria**  **(Annex 1)** | 0 | 1 | 1 | 0 | 1 | 0 | 1 |
| **Sample size adequacy** | 0 | 1 | 0 | 1 | 1 | 1 | 1 |
| **Sample size balance in groups** | 1 | 1 | 1 | 1 | 0 | 1 | 1 |
| **Age balance in groups** | 1 | 1 | 1 | 1 | 1 | 1 | 1 |
| **Sex balance in groups** | 0 | 0 | 1 | 1 | 1 | 1 | 1 |
| **Randomized study** | 1 | 0 | 0 | 0 | 0 | 1 | 1 |
| **Control-intervention group similarity** | 1 | 0 | 1 | 1 | 1 | 1 | 1 |
| **Outcomes under risk of bias** | Mortality | LOS  COST | Readmission | - | - | - | - |

Result of sensitivity analysis

| Outcome | RR or WMD [95% CI] | Sensitivity analysis | | | |
| --- | --- | --- | --- | --- | --- |
|  |  | Excluding the  study with high risk of bias* | Excluding the largest  study | Excluding the smallest  study | Excluding the earliest  study |
| In-hospital mortality | 0.45 [0.21, 0.94] | 0.46 [0.21, 1.01] | 0.33 [0.20, 0.54] | 0.47 [0.20, 1.10] | 0.47 [0.20, 1.10] |
| Re-admission rate | 0.81 [0.66, 0.99] | 0.78 [0.60, 1.01] | 0.68 [0.52, 0.89] | 0.78 [0.60, 1.01] | 0.78 [0.60, 1.01] |
| Length of stay | -1.89 [-2.44, -1.33] | -1.97 [-2.82, -1.12] | -1.97 [-2.73, -1.22] | -1.89 [-2.52, -1.27] | -1.97 [-2.73, -1.22] |
| Cost | -1.57 [-3.66, 0.52] | -0.69 [-1.97, 0.58] | -1.65 [-4.70, 1.40] | -2.35 [-4.11, -0.58] | -1.65 [-4.70, 1.40] |

* According to lowest score from the Annex 3.
